# The effect of mandala coloring on anxiety and quality of life of women in the climacteric period: a randomized controlled study

**DOI:** 10.1590/1806-9282.20240059

**Published:** 2024-07-19

**Authors:** Ayça Şolt Kırca, Elif Dağlı, Efsun Derin, Nurettin Aka

**Affiliations:** 1 Kırklareli University, Faculty of Health Sciences, Department of Midwifery – Kırklareli, Turkey.; 2 Çukurova University, Abdi Sütcü Vocational School of Health Services, Department of Health Care Services – Adana, Turkey.; 3 Kırklareli University – Kırklareli, Turkey.

**Keywords:** Climacteric, Women, Menopause, Painting, Anxiety, Quality of life

## Abstract

**OBJECTIVE:**

This study was conducted to determine the effect of mandala coloring on anxiety and quality of life of women in the climacteric period.

**METHODS:**

This research was conducted as an experimental study based on a randomized controlled pre-test and post-test model (single-blind). The study was conducted with women in the climacteric period who presented to a training and research hospital in a western city of Turkey between November 1, 2022, and April 28, 2023. Participants were divided into mandala coloring (n=38) and control groups (n=38).

**RESULTS:**

According to the women's socio-demographic and descriptive characteristics, mean age, body mass index, and frequency of menopausal symptoms were similar in both groups. The mean post-test scores of the women in the mandala coloring group on the state-trait anxiety inventory and menopause-specific quality of life questionnaire vasomotor, psychosocial, physical, and sexual subscales (29.71±5.22, 0.86±0.97, 0.53±0.61, 0.79±0.84, and 0.92±1.24, respectively) were lower than the mean post-test scores of the women in the control group on the same scales (41.02±1.20, 1.79±1.76, 1.49±1.39, 1.72±1.38, and 1.95±1.82, respectively) (p=0.000).

**CONCLUSION:**

Mandala coloring reduces menopause-related anxiety levels and improves quality of life effectively.

## INTRODUCTION

Effective management of anxiety, which can aggravate menopausal symptoms, may improve the quality of life of women in the climacteric period^1^. Therefore, anxiety management may be effective in helping women cope with menopause symptoms. Many alternative methods have been used in the literature to reduce anxiety (music medicine, acupressure, or virtual reality glasses)^1-5^, and the therapeutic effect of mandala coloring, which is one of the art therapy methods, has been reported in some studies^6,7^. Mandala coloring is a safe and accessible activity that does not require any special skills or training and can be used as a complementary strategy to reduce anxiety^6-8^. In a systematic review, Abbing et al. showed that art therapy had a positive effect on reducing anxiety in patients^9^. Flett et al. indicated that daily mandala coloring helped reduce anxiety^10^.

Art therapy is considered therapeutic in the sense that it reconciles emotional conflicts, increases awareness, reduces anxiety, provides relief from destructive emotions and traumas, creates opportunities to solve problems, directs people to reality, improves social skills, and increases self-esteem. Art therapy is a form of expression that helps people give meaning to their inner world and reflect their unconscious emotions externally. In this respect, it also activates creative problem-solving activities^11-14^.

A review of the literature indicated that there was no study about the effects of mandala coloring on menopausal symptom-related anxiety and quality of life of women in the climacteric period. In this context, this study was conducted to determine the effect of mandala coloring on the anxiety and quality of life of women in the climacteric period.

## METHODS

### Research design

**Figure 1:**
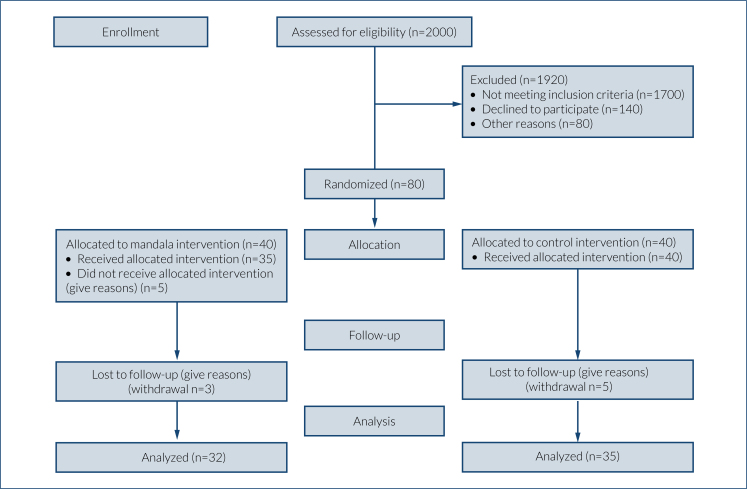
CONSORT flowchart.

An experimental study was conducted based on a randomized controlled pre-test and post-test model. The study was carried out according to CONSORT guidelines (Figure 1), and a clinical trial registration code was obtained (NCT05575349)^15^.

### Participants

The population of the study comprised women who were in the climacteric period and presented to a training and research hospital in a western city of Turkey for outpatient treatment between December 15, 2022, and April 28, 2023. The G*Power software (version 3.1.9.3) was employed to calculate the sample size of the study. A review of the literature indicated that there were no studies on whether mandala coloring reduced the anxiety of women in the climacteric period. The COHEN standard effect size was assumed to be 0.70 to determine the sample size of the study. Accordingly, it was calculated as 68 women (n=34 in each group), based on a Type I error of 0.05, a test power of 0.80 (power analysis) (α=0.05, 1–β=0.80, effect size=0.70), and a 1:1 distribution ratio. Considering some attrition, it was decided to recruit 80 women (n=40 in each group). Participants were randomly assigned to mandala coloring and control groups using the simple random number generator software. This list was recorded by the researcher.

Study inclusion criteria: Women who volunteered to participate in the research, filled out the questionnaires and scales completely, could read and understand Turkish, were in the climacteric period (42–65 years old), and had an STAI score of ≥37 were included in the study.

### Data collection tools

Descriptive information form (DIF): This form was created by the researchers following a review of the literature^5-10^.

The state-trait anxiety inventory (STAI): Spielberger et al. developed this inventory, and Öner and Compte conducted its Turkish adaptation. A high score indicates a high anxiety level, while a small score shows a low level of anxiety. A total score of 36 or less means no anxiety, scores between 37 and 42 indicate slight anxiety, and scores that are greater than 42 show high anxiety^16,17^.

Menopause-specific quality of life questionnaire (MENQOL): Hilditch et al. developed this scale, and Kharbouch and Şahin adapted it into Turkish. High scores from the scale show increased severity of the complaint and decreased quality of life^18,19^.

### Data collection procedure

The first interview with the women was made when they applied to the clinic. To prevent bias in the research, a nurse/midwife who worked at the outpatient clinic that day but was not involved in the study helped the participants fill out the DIF, STAI-S, and MENQOL forms face-to-face during the pre-test phase. Contact information of the women was taken. Post-test data were collected through an online form. It took approximately 20–25 min to fill out the form.

### Interventions

Mandala coloring group: Women were given 12-color felt-tip pen sets and 12 mandala coloring pages by the researcher. They were asked to choose a coloring page and color it as they liked once a week, at any time of the day, for an average of 20–30 min each time. This application took 6 weeks.

Participants were asked to send a message before they started mandala coloring and to take a picture of the coloring and send it to the researcher when they finished the session. They were sent a reminder message twice a week in case they forgot to do mandala coloring.

Control group: The participants in this group comprised people who did not routinely do any practice on their own to reduce their anxiety symptoms. During the same 6 weeks, they were called by the researcher twice a week to find out whether they had taken any action to reduce their anxiety symptoms. Those who used either pharmacological or non-pharmacological practices to reduce anxiety symptoms were excluded from the study. At the end of the study, participants in the control group were informed about mandala coloring, and those who wanted to practice it were given the same chance to do it.

### Data analysis

The Shapiro-Wilk test was employed to check the normality of the variables included in this study. Descriptive statistics and chi-square test were used to compare categorical data. In normally distributed data, the independent-samples t-test was employed to make inter-group comparisons, and the dependent-samples t-test was used to make intra-group comparisons. The Mann-Whitney U test was used to compare data that did not show normal distribution. To present the analysis results, median values (minimum–maximum) were used for non-normally distributed data, mean±standard deviation values were employed for normally distributed data, and frequency (percentage) values were utilized for categorical data. The confidence interval was determined to be less than 0.05.

### Ethical considerations

Ethics committee approval of the research was obtained from the Non-Interventional Clinical Research Ethics Committee of a state university (approval date: October 7, 2022, and decision number: 126/38).

## RESULTS

The comparison of the participants' sociodemographic and descriptive characteristics indicated that mean age, body mass index (BMI), and frequency of menopausal symptoms were similar in both groups. The education level of the women in the experimental group was higher, and the majority of them were in their postmenopausal period for 5 years or more (Table 1).

**Table 1:** Socio-demographic and descriptive characteristics.

		Mandala coloring group (n=32)		Control group (n=35)	
Age		54.31±5.78 (43–65)		53.08±3.92 (44–60)	
		n	%	n	%
BMI					
	Normal	8	25	10	28.6
	Overweight	9	28.1	10	28.6
	Obese	15	46.9	15	42.9
Education status					
	Secondary education	15	46.9	20	57.1
	High school and above	17	53.1	15	42.8
Menopausal period					
	Less than 5 years postmenopause	13	40.6	18	51.4
	5 years or more postmenopause	19	59.3	17	48.5
Frequency of experiencing menopause symptoms					
	Weekly	17	53.1	24	68.5
	Monthly	15	46.9	11	31.4

The anxiety and quality of life levels of women in the mandala coloring and control groups were analyzed using their mean scores on the pre-test and post-test applications of the STAI and MENQOL scales, and intra-and inter-group comparisons were made.

The intra-group comparisons of STAI-S scores of the participants in the mandala coloring group indicated that their mean post-test STAI-S score (29.71±5.22) was lower than their mean pre-test score (42.90±4.29) and that the difference was significant (p<0.001). There was no statistical difference between the mean scores of the participants in the control group from the pre-test and post-test applications of the STAI-S (41.22±1.47 and 41.02±1.20, p>0.05).

The inter-group comparisons of the mean pre-test STAI-S scores indicated that there was no significant difference between mandala coloring (42.90±4.29) and control groups (41.22±1.47) (p>0.05). However, the mean post-test STAI-S score of the participants in the mandala coloring group (29.71±5.22) was significantly lower than that of the participants in the control group (41.02±1.20) (p=0.000) (Table 2).

**Table 2:** Intra-and inter-group comparisons of the mean state-trait anxiety inventory and menopause-specific quality of life questionnaire scores obtained by the participants in the mandala coloring and control groups.

		Mandala coloring group (n=32)	Control group (n=35)	t*	p_2_
STAI-S					
	Pre-test	42.90±4.29	41.22±1.47	t=2.174	p=0.33
	Post-test	29.71±5.22	41.02±1.20	t=-12.458	p=0.000
	t**	t=10.533	t=1.022		
	p_1_	p=0.000	p=0.314		
MENQOL-vasomotor domain					
	Pre-test	1.79±1.76	2.85±0.77	t=-3.242	p=0.002
	Post-test	0.86±0.97	2.92±0.74	t=-9.755	p=0.000
	t**	t=3.154	t=-1.022		
	p_1_	p=0.004	p=0.314		
MENQOL-psychosocial domain					
	Pre-test	1.49±1.39	2.28±0.54	t=-3.121	p=0.003
	Post-test	0.53±0.61	2.32±0.57	t=-12.306	p=0.000
	t**	t=3.727	t=-1.435		
	p_1_	p=0.001	p=0.160		
MENQOL-physical domain					
	Pre-test	1.72±1.38	2.47±0.50	t=-2.993	p=0.004
	Post-test	0.79±0.84	2.48±0.50	t=-10.068	p=0.000
	t**	t=3.797	t=-1.160		
	p_1_	p=0.001	p=0.254		
MENQOL-sexual domain					
	Pre-test	1.95±1.82	3.30±0.83	t=-3.933	p=0.000
	Post-test	0.92±1.24	3.26±0.79	t=-9.036	p=0.000
	t**	t=3.456	t=1.675		
	p_1_	p=0.002	p=0.103		

t*: independent-samples t-test;

t**: dependent-samples t-test.

p_1_intra-group comparisons;

p_2_:inter-group comparisons;

STAI-S, STAI-T: state-trait anxiety inventory;

MENQOL: the menopause-specific quality of life questionnaire.

The intra-group comparison of MENQOL subscale scores indicated that the mean post-test scores of the mandala coloring group from the vasomotor, psychosocial, physical, and sexual subscales (0.86±0.97, 0.53±0.61, 0.79±0.84, and 0.92±1.24, respectively) were lower than their mean pre-test scores from the same subscales (1.79±1.76, 1.49±1.39, 1.72±1.38, and 1.95±1.82, respectively) and that the difference was statistically significant (p<0.05). There was no statistical difference between pre-test and post-test MENQOL vasomotor, psychosocial, physical, and sexual subscale scores in the control group (p>0.05).

The inter-group comparison of MENQOL subscale scores showed that there were differences between the mandala coloring and control groups in terms of their mean pre-test and post-test vasomotor, psychosocial, physical, and sexual subscale scores (p<0.05). It was observed that the mean pre-test and post-test MENQOL subscale scores of the control group were higher than those of the mandala coloring group (Table 2).

## DISCUSSION

The groups in this study were almost similar in terms of sociodemographic and descriptive characteristics (age, BMI, education level, menopausal period, frequency of menopausal symptoms, etc.), which supported the reliability of the study. The results of the study were similar to those of other national and international studies^6,10,20,21^.

In this study, the mean STAI scores of the participants who did mandala coloring decreased significantly compared with those of the control group. In a study conducted with 31 pregnant women, Amelia et al. reported that anxiety scores decreased significantly in women who drew and colored mandalas compared with those of the control group^22^. Yakar et al. conducted a quasi-experimental study with 12 women with breast cancer for 8 weeks and found that the anxiety scores of the participants who received mandala art therapy decreased significantly compared with those before the intervention^23^. In their study with 40 nurses, 37 of whom were female, Maguire et al. reported that mandala coloring for 20 min during breaks reduced their anxiety^24^. Akbulak and Can conducted a quasi-experimental study with 84 participants to examine the effect of mandala coloring on reducing the stress experienced by women with early-stage breast cancer during their first chemotherapy session and found that mandala coloring significantly reduced their anxiety levels^25^. In a quasi-experimental study with 200 patients diagnosed with multiple sclerosis, 55% of whom were female, Barati et al. reported that mandala coloring for 4 weeks significantly reduced anxiety scores^26^.

The increase in vasomotor symptoms and sleep disturbances in menopause potentiates depression, sleep difficulty, decreased sleep quality with advancing age, as well as moderate to severe climacteric symptoms, which can affect cognitive function^27^. Different aspects such as a decline in hormonal levels, vaginal dryness, genital atrophy with pain, and fear of meeting their partners' expectations contribute to the progressive decline in sexual activity^28^. Additionally, depression and vasomotor symptoms may affect these women's sexual activities, their spouses' health problems, or marital problems^29^. In this study, the menopause-specific quality of life of participants who did mandala coloring was evaluated, and MENQOL vasomotor, psychosocial, physical, and sexual subscale scores decreased significantly compared with those of the control group. There was no study in the literature on the examination of the effect of mandala coloring on menopause symptoms in women in the climacteric period, and the results of our study were parallel to the results of studies conducted with alternative methods used to improve the quality of life of women in the climacteric period^26,30^.

The results of many studies conducted to date have supported the application of mandala coloring, which is among the art therapy methods, in the treatment of various health problems. It has been reported in the literature that the effectiveness and reliability of this method have not been reduced by any complications. Mandala, which is an integrative body–mind education method, created a meditative effect with its repetitive patterns and symmetry, increased psychological well-being by developing awareness, and also demonstrated its anxiety-reducing effect. This, in turn, contributed to a significant decrease in STAI and MENQOL subscale scores. Therefore, mandala coloring can be recommended as an effective method to reduce anxiety and menopause-specific symptoms during the climacteric period and improve quality of life.

The results of this randomized controlled trial apply only to the women in this research and cannot be generalized to others. The lack of high-quality RCTs and quasi-experimental studies testing these hypotheses limits the generalization of our results.

## CONCLUSION

Our study data show that mandala coloring reduces mean STAI-S and MENQOL vasomotor, psychosocial, physical, and sexual subscale scores of women in the climacteric period and that this practice is an effective intervention that can be used to alleviate anxiety symptoms in the climacteric period and to improve menopause-specific quality of life. Mandala coloring, which has a therapeutic effect, is recommended to be widely used in such populations.

Mandala painting can be applied in more sessions and its effect can be evaluated. The effect of unstructured mandala coloring and structured mandala coloring can be compared. In addition, by comparing different art therapy methods with each other, the method that is effective in reducing psychological symptoms can be investigated.
